# Severity Profiles of Smartphone Addiction and Mental Health Differences from the Perspective of Adolescent Development: A Latent Profile Analysis

**DOI:** 10.3390/bs16101820

**Published:** 2026-10-04

**Authors:** Na Zhang, Xuemei Gao, Yaxin Yang, Yunrui Wang, Xuan Xia, Yuhong Zhou, Yujie Huang, Zijie Fang

**Affiliations:** 1 Psychological Research and Consultation Center, Southwest Jiaotong University, Chengdu 610031, China; 18301170130@fudan.edu.cn (N.Z.); yangyaxin@my.swjtu.edu.cn (Y.Y.); wangyunrui@my.swjtu.edu.cn (Y.W.); xuan2025212149@my.swjtu.edu.cn (X.X.); zhouyh@swjtu.edu.cn (Y.Z.); 2 School of Marxism, Southwest Jiaotong University, Chengdu 610031, China; yjhuang@my.swjtu.edu.cn (Y.H.); jasonleon@my.swjtu.edu.cn (Z.F.)

**Keywords:** smartphone addiction severity, adolescents, individual differences, latent profile analysis, mental health

## Abstract

Introduction: In the digital age, smartphones may amplify adolescents’ developmental vulnerability, making their mental health more likely to be affected by problematic smartphone use. However, prior research has largely overlooked adolescents’ developmental characteristics and individual differences. Methods: This study employed latent profile analysis (LPA) to identify distinct severity profiles of smartphone addiction risk among 2158 Chinese adolescents (53.1% boys; mean age = 14.07 years). We further examined the associations of demographic variables with profile membership, and applied the BCH weighting method to compare differences in well-being, anxiety, and depression across profiles. Results: (1) adolescent smartphone addiction could be classified into three severity profiles: a well-adjusted use group (31%), a low-risk group (53%), and a high-risk group (16%); (2) age, grade, and social economic status were significantly associated with profile membership; (3) the three smartphone addiction profiles differed significantly in concurrently measured mental health outcomes, with higher addiction risk associated with lower well-being and higher levels of anxiety and depression. Conclusions: Adolescent smartphone addiction reflects categorized severity and the profiles show graded cross-sectional differences in mental health. Future research should consider adolescent developmental characteristics and employ longitudinal designs to validate these severity profiles.

## 1. Introduction

Adolescence is a critical developmental period marking the transition from childhood to adulthood and it is also a high-risk window for mental health problems (Orben et al., 2022; Solmi et al., 2022). This period is characterized by rapid physical and psychological changes and face challenges across behavior, cognition, and neurodevelopment domains. Behaviorally, adolescents show heightened sensation seeking, while self-regulation is still maturing (Chein et al., 2011; Steinberg et al., 2018). Cognitively, adolescents become increasingly concerned about social evaluation, and their tendency toward social comparison and sensitivity to peer feedback grows stronger (Crone et al., 2022; Dahl et al., 2018). At the neurodevelopmental level, the reward system is highly sensitive, whereas the brain regions responsible for cognitive control and emotion regulation are not yet fully mature (Braams et al., 2015; Crone & Konijn, 2018). Thus, even before the rise of smartphones, adolescence itself already involved developmental features that made it a sensitive period and a vulnerable window for mental health.

In the digital age, smartphones may strengthen these developmental vulnerabilities. Smartphones do not directly affect adolescents’ mental health in isolation; rather, their effects are shaped by interactions with adolescents’ behavioral, cognitive, and neurodevelopmental characteristics. Uses and gratifications theory (UGT) holds that individuals are not passive receivers of media and they actively choose media to meet their needs (Sun & Zhang, 2021). For adolescents, smartphones now serve social interaction, entertainment, education, and information seeking (Bar et al., 2025). Compared with adults, adolescents place greater value on peer relationships and are more sensitive to others’ evaluations. Smartphones provide opportunities for social interaction, self-presentation, and peer feedback. Moreover, the quantifiable, readily accessible, and variable-reward features of smartphones and their affiliated platforms align closely with developmental sensitivities during adolescence (Orben et al., 2024). The dual systems model helps explain these tendencies from a developmental view. In this model, the socioemotional system that responds to reward matures earlier than the cognitive control system that supports self-regulation (Shulman et al., 2016; Steinberg et al., 2018). This imbalance helps explain why the variable-reward and rapid-feedback features of smartphones attract adolescents and why some find it hard to control their use, potentially leading to smartphone addiction and difficulties in emotion regulation (X. Ji et al., 2025; Wu-Ouyang, 2022). It is important to note that smartphone addiction is not currently recognized as a formal clinical diagnosis in major diagnostic manuals such as the DSM-5 or ICD-11. In this study, we conceptualize smartphone addiction as behavioral addiction tendency or problematic smartphone use (PSU), defined as excessive and compulsive use that leads to functional impairment (Lin et al., 2014).

Adolescents’ unique neurodevelopmental features also provide a biological vulnerability for the development of problematic smartphone use. The Interaction of Person–Affect–Cognition–Execution (I-PACE) model posits that smartphone addiction does not arise from digital technology alone, but emerges from dynamic interactions among individuals’ emotional and cognitive processes, executive control functions, and the media environment (Brand et al., 2019). Some adolescents use smartphones as a strategy for escaping stress and regulating negative emotions and this pattern can become a vicious cycle in which greater dependence leads to greater anxiety, and greater anxiety leads to greater dependence (Brand et al., 2025; Kardefelt-Winther, 2014). For some adolescents, high-frequency use may progress into problematic smartphone use, a pattern of increasingly compulsive use characterized by loss of control and continued use despite negative consequences (Hollis, 2022; Nagata et al., 2026). These ideas offer a useful background for interpreting why adolescents with higher smartphone addiction risk may report lower well-being and higher anxiety and depression. However, the present study did not directly measure the emotional, cognitive, executive control, or media environment processes described by I-PACE, so these explanations are treated as possibilities rather than tested mechanisms. Compared with children and adults, adolescents show a particularly clear vulnerability to problematic smartphone use. Examining the link between smartphone addiction risk and mental health therefore requires treating adolescents as a distinct population that warrants focused investigation.

It also suggests individual differences, because the pace of development of two systems varies across adolescents, so their smartphone use and its link to mental health may vary as well. Latent profile analysis (LPA) can identify severity profiles of smartphone addiction risk and better capture behavioral differences within the adolescent population, as well as their corresponding mental health profiles (Oberski, 2016; Stanley et al., 2017). However, most studies on smartphone addiction risk have focused on college students or adults. Although a few studies have examined adolescents, they often lack a theoretical basis grounded in adolescent developmental characteristics. Therefore, they do not fully explain why heterogeneity exists or how different profiles reflect distinct developmental features. Moreover, studies examining the relationship between smartphone addiction risk and mental health often address only a single outcome, such as well-being, anxiety, or depression (Lo Coco et al., 2020; Zhou et al., 2021).

Based on the above background, the present study used LPA in a large sample of adolescents aged 12 to 18 years to identify distinct patterns of smartphone addiction risk. Based on adolescent developmental characteristics, we examined the features of these profiles from an individual-differences perspective. We also explored group differences in depression, anxiety, and well-being, and tested how demographic variables predict profile membership.

Specifically, this study aimed to address the following questions:

- RQ1: What distinct profiles of smartphone addiction exist among adolescents aged 12 to 18 years, and what are the distributional characteristics of these profiles across smartphone addiction indicators?
- RQ2: Do sex, age, and social socioeconomic status predict adolescents’ membership in specific smartphone addiction profiles?
- RQ3: Do adolescents with different smartphone addiction profiles differ significantly in well-being, anxiety, and depression?

Based on the I-PACE and UGT theoretical framework and empirical evidence reviewed above, we proposed the following hypotheses:

**H1.**  *Grounded in the I-PACE model, which emphasizes individual differences in susceptibility and person–environment interactions, we hypothesize that adolescents can be classified into distinct profiles representing different levels of smartphone addiction risk, such as well-adjusted, low-risk, and high-risk smartphone addiction group.*

**H2.**  *Based on the UGT, which highlights diverse usage motivations, we hypothesize that demographic variables significantly predict profile membership. Specifically, girls, older adolescents, and those with lower socioeconomic status are expected to be more likely to belong to higher-risk profiles.*

**H3.**  *Consistent with the I-PACE model linking excessive technology use to emotional impairments, we hypothesize that adolescents in higher-risk profiles will report lower levels of well-being and higher levels of anxiety and depression compared to those in low-risk profiles.*

## 2. Methods

### 2.1. Participants

Participants were recruited from one primary school, one junior high school, and one senior high school in a city in China. We employed a stratified cluster random sampling method, so we randomly selected several classes from different grades to distribute the questionnaires. A total of 2300 adolescents were invited to complete the questionnaires. During the double-entry data preprocessing, questionnaires were excluded if they had more than 3 missing items or selected the same option for 3 consecutive items. Consequently, 2158 valid questionnaires were retained, yielding a valid response rate of 93.83%. Missing data in the retained questionnaires were minimal and handled using Full Information Maximum Likelihood (FIML). The final sample consisted of 1146 boys (53.1%) and 1012 girls (46.9%), with a mean age of 14.07 years (SD = 1.80). The sample included upper primary school students, middle school students, and high school students, with ages ranging from 12 to 18 years, thus broadly representing the adolescent population.

### 2.2. Measures

#### 2.2.1. Smartphone Addiction Scale

Smartphone addiction was measured using the Smartphone Addiction Scale—Short Version (SAS-SV) developed by Kwon (Kwon et al., 2013). The SAS-SV has shown good reliability and validity in assessing smartphone addiction (J. Li et al., 2023). The scale contains 10 items covering the core behavioral features of smartphone addiction, including daily life disturbance, withdrawal symptoms, and difficulty controlling use. Participants responded on a 6-point Likert scale ranging from 1 (strongly disagree) to 6 (strongly agree). The Chinese version of this scale has demonstrated good reliability and validity and the Cronbach’s alpha was 0.855 in previous studies (Xiang et al., 2019). In this study, the Cronbach’s alpha was 0.889.

#### 2.2.2. Well-Being Scale

Well-being was measured using the Campbell Index of Well-Being developed by Campbell (Campbell, 1976). This scale is mainly used to assess participants’ perceived level of well-being across two dimensions: general affect index and life satisfaction. The scale consists of 9 items rated on a 7-point Likert scale, with higher scores indicating greater well-being. The Cronbach’s alpha of Chinese version was 0.780 in previous studies (L. Li & Shen, 2009). In this study, the Cronbach’s alpha coefficient was 0.876.

#### 2.2.3. Anxiety Scale

Anxiety symptoms were assessed using the 7-item Generalized Anxiety Disorder scale (GAD-7; Spitzer et al., 2006), a widely used screening tool for generalized anxiety disorder and symptom severity. Total scores range from 0 to 21, with higher scores reflecting more severe anxiety. The Cronbach’s alpha of Chinese version was 0.898 in previous studies (Su et al., 2022). In this study, the Cronbach’s alpha coefficient was 0.904.

#### 2.2.4. Depression Scale

Depressive symptoms were measured using the 10-item Center for Epidemiological Studies Depression Scale (CES-D-10; Andresen et al., 1994), a brief version of the original CES-D that has been widely used in large-scale surveys. Items are rated on a 4-point scale from 0 (rarely or none of the time) to 3 (most or all of the time), with total scores ranging from 0 to 30. Higher scores indicate more severe depressive symptoms. The Cronbach’s alpha of Chinese version was 0.815 in previous studies (Huang et al., 2015). In this study, the Cronbach’s alpha coefficient was 0.835.

### 2.3. Statistical Analysis

Descriptive statistics, correlation analyses, and tests of common method bias were conducted using SPSS 26.0. LPA was performed using Mplus 8.3 with the robust maximum likelihood estimator (MLR). Missing data were handled using Full Information Maximum Likelihood (FIML). Models with two through five profiles were estimated using 200 random starts and 50 final-stage optimizations. Indicator variances and covariances were freely estimated across profiles.

The data analysis proceeded in three steps. First, LPA was conducted to identify smartphone addiction risk profiles. Models with two through five profiles were estimated, and the optimal number of profiles was determined based on a combination of statistical fit indices and conceptual interpretability. Model fit was evaluated using the following criteria: (1) information criteria, including the Akaike Information Criterion (AIC), Bayesian Information Criterion (BIC), and sample-size adjusted BIC (aBIC), with lower values indicating better fit; (2) entropy, which reflects classification accuracy, with values closer to 1.0 indicating more precise classification; and (3) likelihood ratio tests, including the Lo–Mendell–Rubin adjusted likelihood ratio test (LMR) and the bootstrap likelihood ratio test (BLRT), for which a significant *p* value suggests that the k-profile model fits better than the k-1-profile model (Lubke & Muthén, 2005). Second, after identifying the optimal profile solution, the R3STEP command in the robust three-step approach was used to examine the logistic regression effects of demographic variables on adolescents’ latent profile membership. VIF values were checked and were below 5, indicating acceptable multicollinearity. Third, the BCH weighting method was applied to compare differences in mental health outcomes across profiles. Hedges’ g and 95% confidence intervals were calculated to evaluate practical significance.

### 2.4. Common Method Bias

Given that all data were collected via self-report questionnaires, common method bias was assessed using Harman’s single-factor test. The results revealed seven factors with eigenvalues greater than 1.0, and the first factor accounted for 30.35% of the total variance, which is below the recommended threshold of 40%. However, because Harman’s single-factor test is relatively insensitive, the potential for common method variance remains an unresolved limitation.

## 3. Results

### 3.1. Descriptive Statistics and Correlations

**Figure 1:**
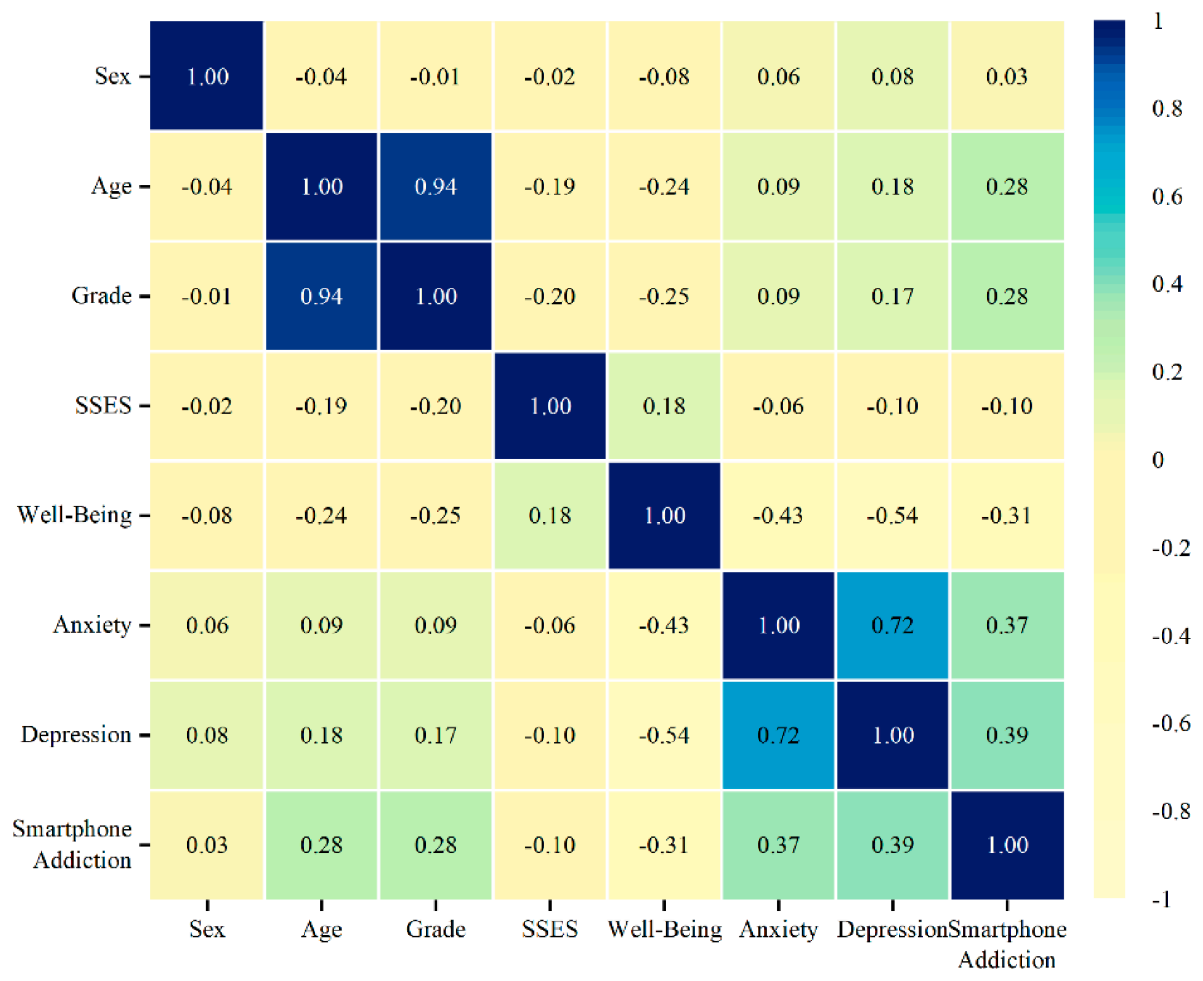
Heatmap of Pearson correlations among study variables. Note. Cell values represent Pearson correlation coefficients; darker shading indicates stronger associations.

Descriptive statistics, including minimum, maximum, mean, standard deviation and correlation analyses for the study variables are presented in Table 1, and the Pearson correlation heatmap is presented in Figure 1. The absolute values of skewness (ranging from 0.076 to 0.965) and kurtosis (ranging from 0.014 to 0.621) were all within the acceptable thresholds (skewness < 1, kurtosis < 1), indicating that all variables were approximately normally distributed. Smartphone addiction risk was significantly negatively correlated with well-being, and significantly positively correlated with anxiety and depression.

### 3.2. Latent Profile Analysis of Smartphone Addiction Risk

To explore the extent of differences in adolescent smartphone addiction risk, the item scores of the SAS-SV scale were entered as indicator variables into the profile model. Models with two to five profiles were compared, and the fit indices are reported in Table 2. Although the AIC, BIC, and aBIC continued to decrease from the two-profile to the five-profile solutions, the magnitude of the decrease diminished substantially after the three-profile model. The three-profile model also yielded the highest entropy value (0.868), indicating good classification accuracy. In addition, both the LMR and BLRTs were significant for the three-profile model (*p* < 0.001), suggesting that the three-profile solution provided a significantly better fit than the two-profile solution. In three profiles, each profile accounted for more than 10% of the sample. Considering the principle of parsimony, classification quality, and theoretical interpretability, the three-profile model was selected as the optimal solution.

**Figure 2:**
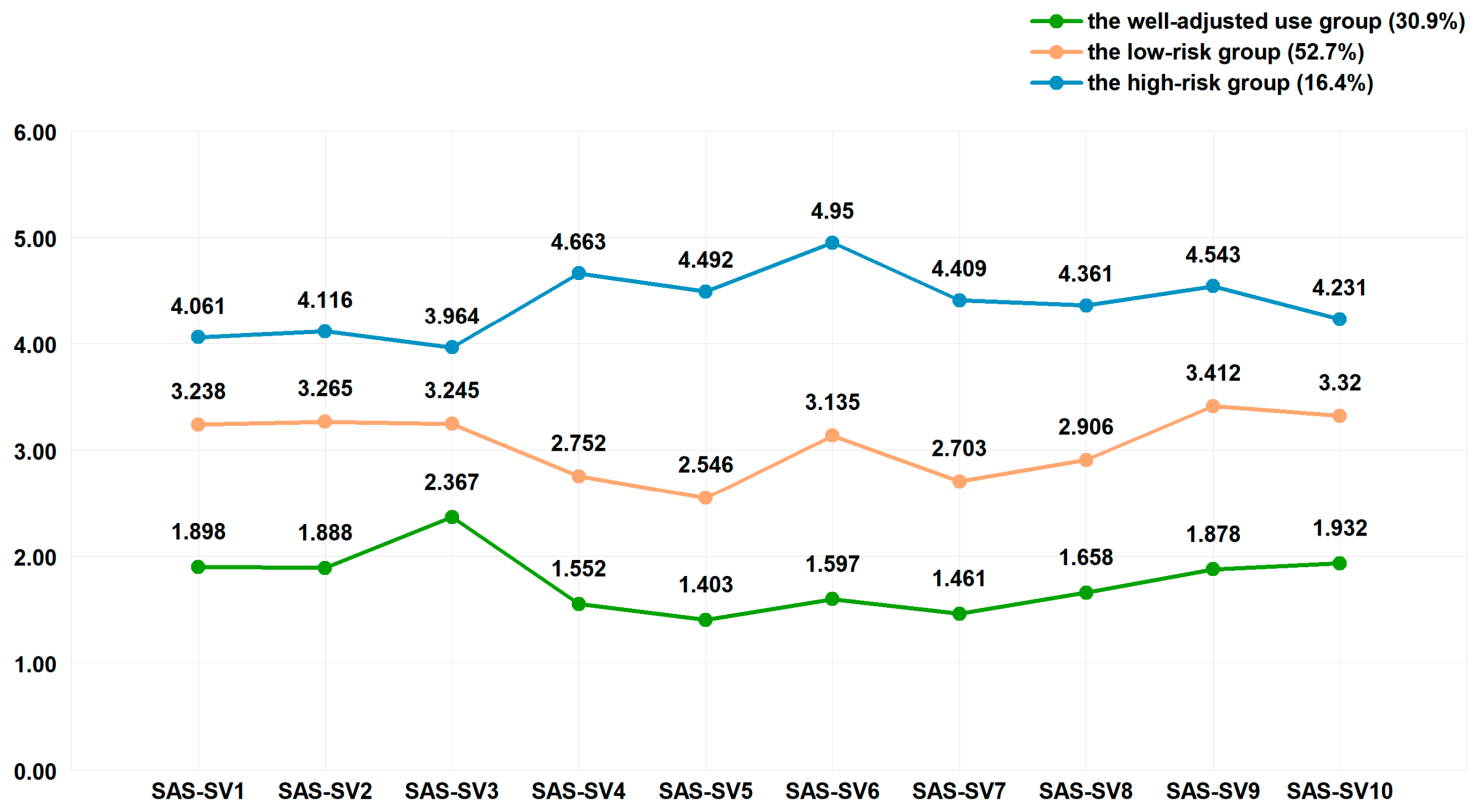
Item-level scores across latent profiles of adolescent smartphone addiction risk.

The score patterns of the three latent profiles across the smartphone addiction items are displayed in Figure 2. The latent profiles did not cross on any item, and the shape trends across the three profiles were generally consistent, indicating differences primarily in overall severity rather than symptom configurations. Profile 1 comprised 666 participants who scored relatively low on all smartphone addiction items; this group was labeled the well-adjusted smartphone use group. Profile 2 included 1137 participants and was labeled the low-risk group. Profile 3 consisted of 355 participants who scored relatively high on all items; this group was labeled the high-risk group.

### 3.3. Demographic Predictors of Smartphone Addiction Profile Membership

**Figure 3:**
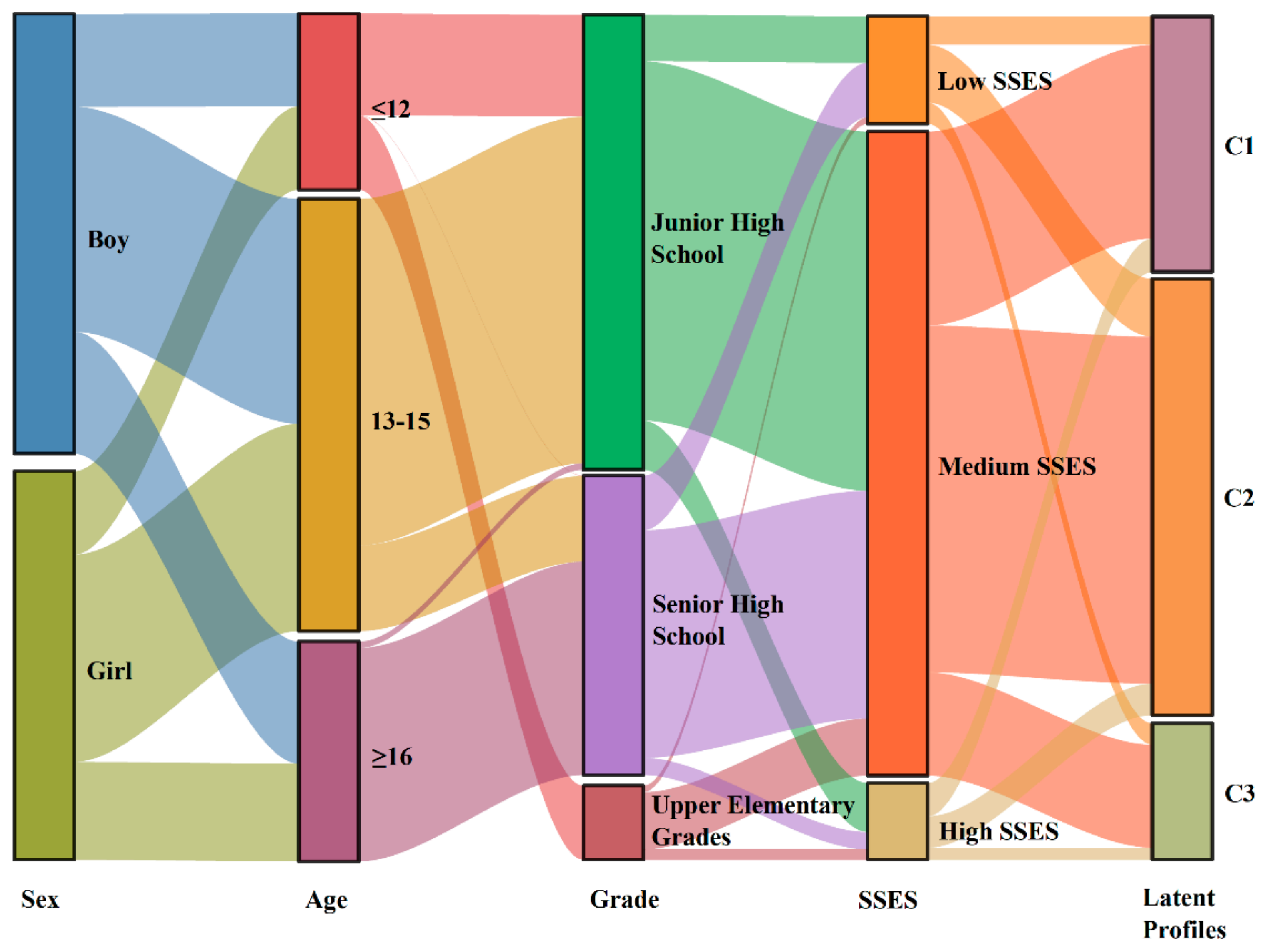
Sankey diagram of demographic characteristics across adolescent smartphone addiction profiles. Note: Flow widths are proportional to the number of adolescents in each category. C1 = the well-adjusted group; C2 = the low-risk group; C3 = the high-risk group.

To examine the effects of demographic variables on profile membership, logistic regression analyses were conducted using the R3STEP approach, with the well-adjusted smartphone use group as the reference category. Predictors included sex, grade, age, and socioeconomic status. A Sankey diagram illustrating the relationships between demographic variables and profile membership is presented in Figure 3. Odds ratios (OR) and their 95% confidence intervals (CI) are reported in Table 3.

The regression results revealed the following patterns. (1) Sex did not significantly predict profile membership. Boys and girls did not differ significantly in their likelihood of belonging to either the low-risk or high-risk smartphone addiction group relative to the well-adjusted use group. (2) Grade and age were highly correlated (r = 0.94). Sensitivity analyses entering them separately yielded consistent results, supporting the robustness of their concurrent associations. For grade, compared with the well-adjusted use group, students in higher grades were more likely to be classified into the low-risk (OR = 1.465, 95% CI [1.359, 1.578], *p* < 0.001) and high-risk (OR = 1.758, 95% CI [1.594, 1.939], *p* < 0.001) groups. Similarly, age was concurrently associated with membership in the low-risk (OR = 1.374, 95% CI [1.283, 1.472], *p* < 0.001) and high-risk (OR = 1.618, 95% CI [1.481, 1.766], *p* < 0.001) groups. (3) Socioeconomic status was a significant negative predictor of profile membership. Higher socioeconomic status scores were associated with lower odds of being classified into the low-risk and high-risk groups relative to the well-adjusted use group. This finding suggests that higher socioeconomic status served as a protective factor against smartphone addiction risk.

In summary, higher grade and older age were risk factors for adolescent smartphone addiction, whereas higher social economic status was a protective factor.

### 3.4. Differences in Mental Health Outcomes Across Smartphone Addiction Risk Profiles

**Figure 4:**
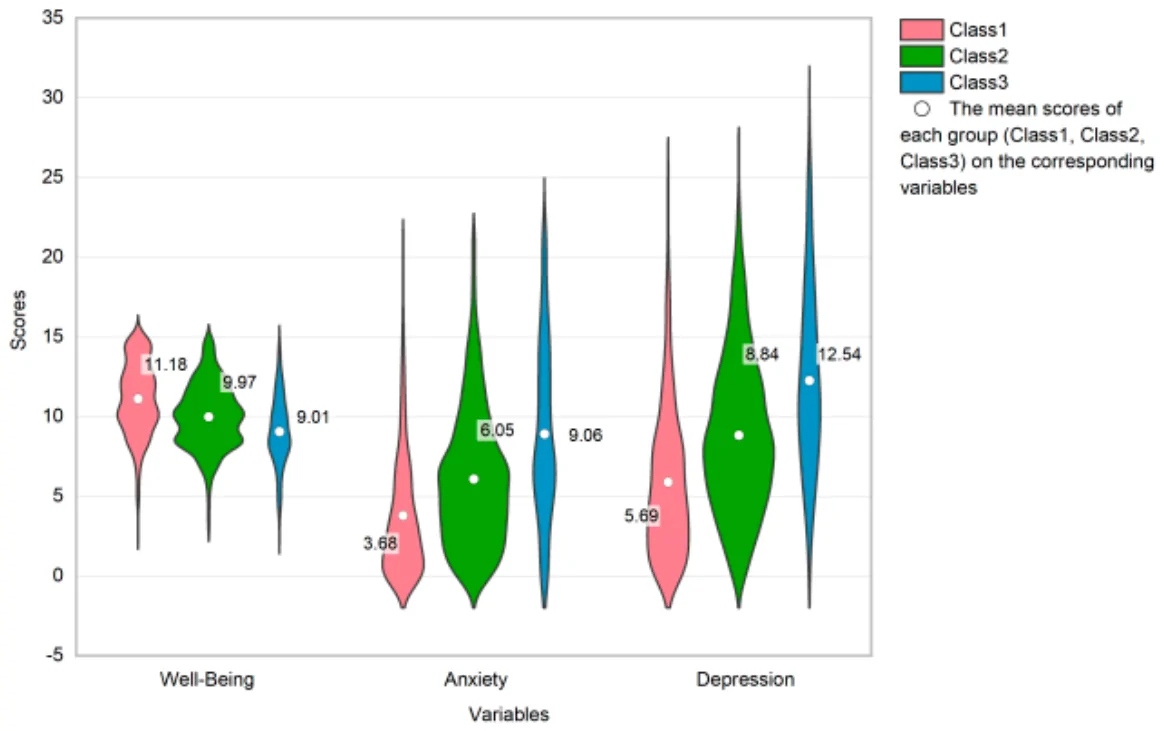
Violin plots of mental health outcomes across three smartphone addiction severity profiles. Note: Class 1 = well-adjusted use group; Class 2 = low-risk group; Class 3 = high-risk group.

**Figure 5:**
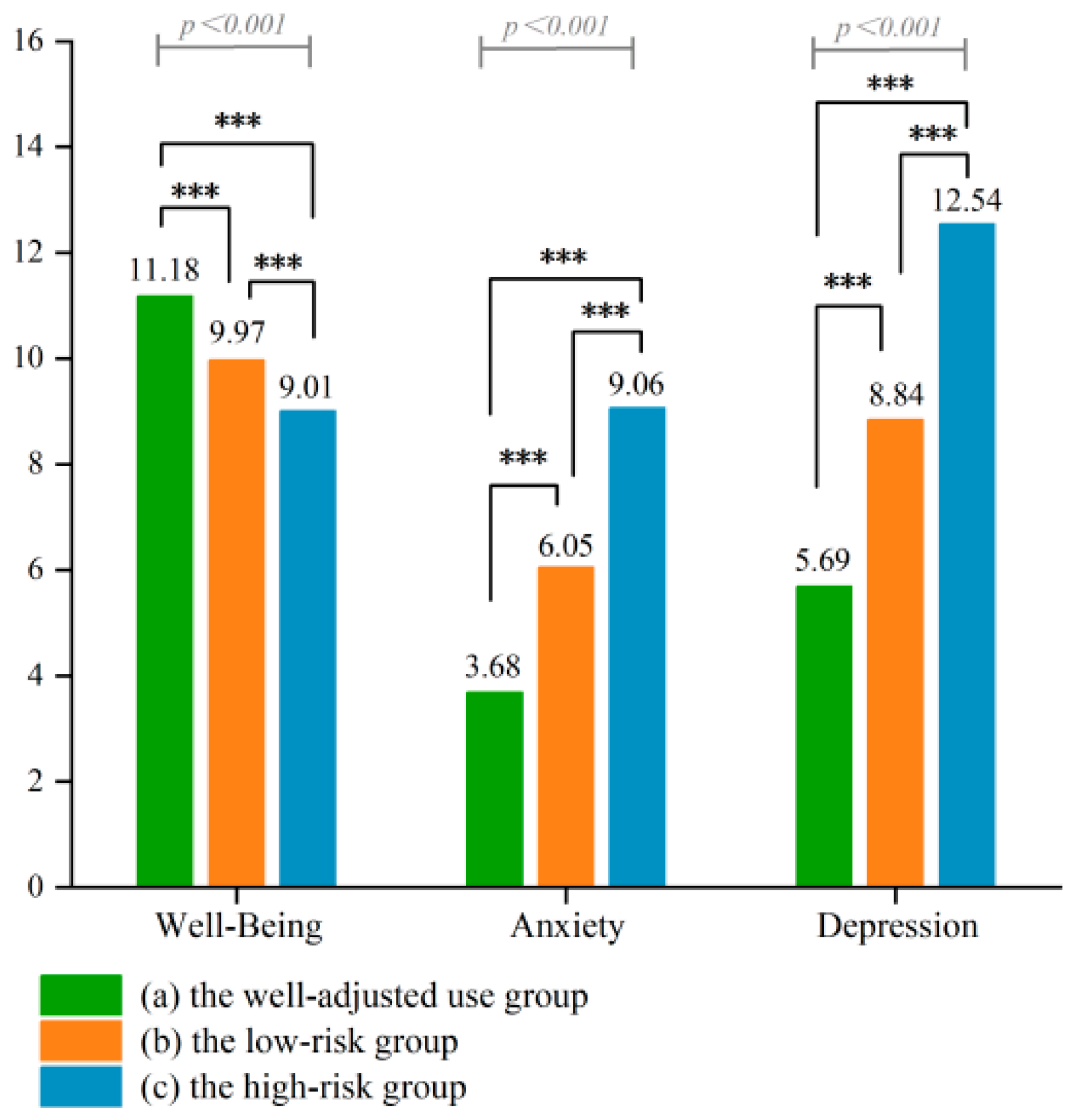
Mean differences in mental health outcomes across adolescent smartphone addiction risk profiles. Note: Brackets indicate pairwise comparisons. *** *p* < 0.001.

To examine the associations between smartphone addiction profiles and mental health, the BCH weighting method was used to compare the three profiles on well-being, anxiety, and depression. Results are presented in Table 4 and visualized in Figure 4 and Figure 5.

Significant differences emerged across the three profiles for all mental health indicators (*p* < 0.001). As illustrated in Figure 4, the violin plots display the full distribution of well-being, anxiety, and depression across the three profiles, providing a more comprehensive view of the data beyond means alone. Specifically: (1) For well-being, the omnibus Wald test was significant (Wald χ^2^(2) = 208.364, *p* < 0.001). The well-adjusted use group reported the highest well-being (M = 11.184), followed by the low-risk group (M = 9.967), and the high-risk group reported the lowest well-being (M = 9.008). All three groups differed significantly from one another (*p* < 0.001), with moderate to large effect sizes (Hedges’ g = 0.45 to 0.91). As illustrated in Figure 5, well-being decreased progressively with increasing smartphone addiction risk, following the pattern of well-adjusted use group > low-risk group > high-risk group. (2) For anxiety, the omnibus Wald test was also significant (Wald χ^2^(2) = 265.463, *p* < 0.001). The high-risk group reported the highest anxiety levels (M = 9.064), followed by the low-risk group (M = 6.053), whereas the well-adjusted use group reported the lowest anxiety (M = 3.675). All pairwise comparisons were significant (*p* < 0.001), with moderate to large effect sizes (Hedges’ g = −0.55 to −1.12). (3) For depression, the omnibus Wald test was significant (Wald χ^2^(2) = 352.628, *p* < 0.001). The high-risk group exhibited the highest depression scores (M = 12.539), followed by the low-risk group (M = 8.839), and the well-adjusted use group reported the lowest depression (M = 5.687). All pairwise comparisons were significant (*p* < 0.001), with moderate to large effect sizes (Hedges’ g = −0.61 to −1.24).

Taken together, these findings indicate that adolescents with higher smartphone addiction risk reported significantly lower well-being and significantly higher levels of anxiety and depression. The graded pattern across the three profiles indicates significant graded associations between smartphone addiction severity and mental health outcomes, though these screening scores do not constitute clinical diagnoses.

## 4. Discussion

Based on a sample of 2158 adolescents aged 12 to 18 years, this study employed latent profile analysis to identify distinct severity levels of smartphone addiction risk and examined their associations with mental health outcomes. First, adolescent smartphone addiction could be classified into three distinct profiles: a well-adjusted use group (30.9%), a low-risk group (52.7%), and a high-risk group (16.4%). Second, age, grade level, and socioeconomic status significantly predicted profile membership, whereas sex did not. Third, the three profiles differed significantly in well-being, anxiety, and depression, with higher addiction risk associated with lower well-being and elevated anxiety and depression. These findings support the view that adolescent smartphone addiction is obvious differences and closely linked to mental health, and they further highlight the importance of adopting a developmental perspective when examining smartphone addiction risk during adolescence.

### 4.1. Profile Characteristics of Adolescent Smartphone Addiction

This study identified three severity profiles of adolescent smartphone addiction risk, well-adjusted use, low-risk, and high-risk, that differed clearly in their scores across smartphone addiction indicators, supporting Hypothesis 1. This finding suggests that adolescent smartphone addiction risk reflects categorized severity levels. (Ding et al., 2024; Kabadayi, 2024). It is important to note that because the profiles were parallel, the LPA primarily categorized a continuous underlying severity dimension. While a continuous-score model might yield similar mental health ordering, the three-profile solution provides a practical descriptive summary by identifying the absolute proportions of adolescents at different risk levels.

Adolescence is a developmental period marked by individual differences. The social interaction, self-presentation, and immediate feedback features of smartphones may interact with adolescents’ heightened reward sensitivity and still-developing cognitive control abilities, leading to diverse patterns of smartphone use (Orben et al., 2024). In this study, the low-risk smartphone addiction group comprised the largest proportion of the sample (52.7%), whereas the high-risk group accounted for a relatively smaller proportion (16.4%). Despite its smaller size, adolescents in the high-risk group scored markedly higher on all addiction indicators. These profiles reflect relative differences in self-reported symptom severity within our sample.

According to the I-PACE model and UGT as explanatory frameworks, adolescents in the high-risk group may experience unmeasured factors such as higher impulsivity, weaker self-regulation, or greater real-life stress (M. Ji et al., 2025). However, these remain untested hypotheses for future research. The well-adjusted use group scored the lowest on all indicators. While they may possess better self-regulation or offline resources, this study cannot confirm these mechanisms. For them, smartphones may remain primarily functional communication tools. They are able to meet their needs for information, social connection, and entertainment while maintaining regulation over their smartphone use. This pattern aligns with findings from previous research suggesting that a subset of adolescents can engage with digital media in a balanced and non-problematic manner (Finserås et al., 2026).

The low-risk group, which constituted the largest proportion, scored slightly lower than the high-risk group. In the cross-sectional data, this group exhibited low levels of self-reported symptoms. Smartphones can serve as useful tools but may also become a coping strategy when emotional regulation and social support are insufficient (Chang & Ko, 2023). Within this cross-sectional sample, the low-risk group exhibited intermediate levels of self-reported symptoms. Whether most adolescents follow a transitional developmental phase over time cannot be determined from these data and requires future longitudinal investigation. The proposition that this pattern reflects underlying neurodevelopmental vulnerability remains an untested hypothesis (Ihm, 2018). The I-PACE model and UGT suggest that unmet developmental needs may relate to different levels of addiction risk, but these mechanisms were not directly tested here.

In sum, this study identified three severity profiles of adolescent smartphone addiction risk. These results highlight the importance of considering developmental perspectives when examining smartphone addiction risk, though longitudinal replication is needed to validate these profiles. From this perspective, adolescence also represents a critical window for early intervention and prevention of smartphone addiction.

### 4.2. Demographic Predictors of Smartphone Addiction Profile

The logistic regression results indicated that age, grade level, and socioeconomic status were significantly associated with membership in different smartphone addiction risk profiles, whereas sex did not. These findings partially support Hypothesis 2.

In the cross-sectional data, both older age and higher grade were positively associated with being classified into the high-risk group, consistent with previous meta-analytic findings (Wei et al., 2024). Age and grade level were significantly associated with higher-risk profile membership. Due to their high overlap, these two variables should be interpreted as jointly reflecting shared developmental, rather than as independent effects. These differences may reflect cohort effects, school-stage transitions, and contextual factors rather than developmental changes within individuals. As students advance to higher grades, increased autonomy, greater smartphone access, and heavier academic stress may co-occur with higher risk (Dwyer et al., 2023; Wang et al., 2026). However, because age and grade were highly correlated and not followed longitudinally, these findings represent cross-sectional associations and cannot imply that risk increases as adolescents grow older.

Second, socioeconomic status emerged as a significant inverse correlate of smartphone addiction risk. Adolescents with higher SSES were less likely to be classified into the high-risk profiles. This inverse association may be explained by unmeasured confounding factors, such as parenting practices, family resources (Hong et al., 2019; Lai et al., 2022). Social economic status was measured with a single subjective item, and possible bidirectionality cannot be ruled out.

In addition, the present study found that sex did not significantly predict latent smartphone addiction risk profile membership. This suggests that, in the digital age, smartphones have become deeply integrated into adolescents’ daily lives, and both boys and girls are exposed to similar usage environments and situational triggers (Holly et al., 2023). This may be because smartphones are no longer merely entertainment devices and they now serve multiple functions, including gaming, social networking, and short-video consumption. Consequently, both boys and girls may develop frequent and uncontrolled use patterns, albeit through different content pathways and for different underlying reasons. Our findings suggest that future research should focus on specific usage motives and behavioral patterns rather than inferring risk from sex alone.

Taken together, these findings highlight that demographic characteristics are concurrently associated with smartphone addiction risk profiles.

### 4.3. Differences in Mental Health Across Smartphone Addiction Profiles

This study found significant differences in well-being, anxiety, and depression across three smartphone addiction risk profiles. The results revealed significant graded cross-sectional associations. Specifically, the higher-risk groups reported lower well-being and higher levels of anxiety and depression. These findings support Hypothesis 3. It reflects a continuous and progressively worsening relationship between smartphone use and mental health as risk accumulates (Kim & Oh, 2026; Weiss & Bonell, 2025). However, because the data are cross-sectional, these differences should be interpreted as concurrent associations rather than causal effects.

In terms of well-being, the well-adjusted group reported the highest level, whereas the low-risk and high-risk groups reported significantly lower well-being. This cross-sectional difference suggests that adolescents’ subjective well-being may be concurrently associated with different levels of smartphone use. These differences may reflect unmeasured mechanisms. For example, future research could test whether adolescents in the higher-risk groups spend more time on their phones, potentially displacing real-life social interaction and offline activities (Angelini et al., 2026; De Lise et al., 2024). In contrast, future studies could examine whether higher-risk adolescents allow smartphone use to occupy time that might otherwise be spent on social interaction, potentially distancing them from real-world support systems (Xu et al., 2025). Positive experiences that might arise from offline activities may be replaced by digital interactions, which could potentially relate to lower well-being (Heinrich et al., 2025).

In the cross-sectional data, adolescents in the higher-risk groups also reported greater anxiety and depression. According to the I-PACE model and compensatory internet use theory, as a theoretical framework for interpretation, adolescents facing negative emotions might turn to smartphones for immediate relief, which could reinforce habitual use (Brailovskaia, 2024; Tao et al., 2023). However, these mechanisms were not measured in this study, and the pathways remain speculative. Future research is needed to test whether excessive smartphone use is associated with reduced social support, shortened sleep, and increased emotional difficulties, which may help explain the concurrent associations observed here (Chen et al., 2019; Shen et al., 2024; Zhao & Shapka, 2026). It is worth emphasizing that the differences in well-being, anxiety, and depression across profiles reflect whether adolescents are successfully completing the developmental tasks (Zhao & Shapka, 2026). Adolescents in the well-adjusted use group may maintain relatively healthy offline relationships and developmental balance, whereas those in the high-risk group show clear digital dependence and adjustment difficulties, and therefore have poorer mental health.

It is noteworthy that although the low-risk group showed lower anxiety and depression than the high-risk group, their mental health levels were still significantly worse than those of the well-adjusted use group. This suggests that mental health risks among adolescents are concurrently associated with varying levels of addiction risk, not only severe addiction (Ren et al., 2025). The low-risk group is not a safe state, rather, since adolescence itself is a period of rapid change and high plasticity (Sydnor et al., 2023). Even if adolescents do not meet the threshold for smartphone addiction, their mental health can still be affected by smartphone use. Consequently, while early attention to lower-risk groups may be beneficial, this proposition requires longitudinal validation.

### 4.4. Implications and Limitations

By confirming the severity levels of smartphone addiction in a large adolescent sample, this study characterized distinct addiction features across different risk groups. This provides a more nuanced perspective for understanding the relationship between adolescent smartphone addiction and mental health. The findings also have implications for future prevention efforts. While this study did not test any intervention, the identification of three distinct severity profiles suggests that tiered intervention strategies may be a valuable proposition requiring longitudinal validation. Specifically, adolescents in the high-risk group, who reported higher levels of addiction symptoms and mental health difficulties, might benefit from targeted support if future research confirms these patterns. Additionally, the low-risk group, which exhibited intermediate levels of self-reported symptoms, may warrant attention to prevent potential escalation over time. Third, schools and families may consider paying attention to adolescents in higher grades and those with lower socioeconomic status, although these cross-sectional associations require cautious interpretation and cannot imply causal protection.

However, this study still has several limitations. First, the cross-sectional design limits causal inference, so the temporal direction between smartphone addiction and mental health cannot be determined. Future studies should use longitudinal tracking or experimental designs to further examine this relationship. Second, the variables in this study were mainly measured through self-report questionnaires, which may be affected by social desirability bias. Additionally, the profile labels reflect relative differences in self-reported scores within our sample and have not been validated against clinical diagnoses. Furthermore, because specific school and class identifiers were not collected due to privacy protections, we could not calculate intraclass correlations or use multilevel models. This means the significance tests should be interpreted with caution, as they do not account for potential clustering effects. Finally, although the sample size was relatively large, participants were recruited from schools in only one region. Therefore, the generalizability of the findings needs to be further tested in broader cultural and regional contexts.

## 5. Conclusions

This study examined the severity levels of smartphone addiction and their associations with mental health among 2158 Chinese adolescents aged 12 to 18 years. Three distinct severity profiles were identified: a well-adjusted use group, a low-risk group, and a high-risk group. Age and grade level were significantly associated with membership in higher-risk profiles, whereas socioeconomic status was inversely associated with profile membership. Sex did not significantly predict profile membership. In the cross-sectional data, significant graded differences were observed: well-being scores were lower, and anxiety and depression scores were higher, across the well-adjusted use, low-risk, and high-risk groups. These findings represent concurrent associations between smartphone addiction severity and mental health.

Collectively, the results underscore the importance of acknowledging different severity levels of adolescent smartphone addiction and suggest that a tiered intervention approach may be a valuable proposition requiring longitudinal validation. Future research is needed to confirm the temporal relationships and clinical significance of these profiles.

## Figures and Tables

**Table 1:** Descriptive statistics and correlations among study variables.

Variable	M	SD	Min/Max	1	2	3	4
1. Smartphone Addiction Risk	28.8	10.04	10/60	——			
2. Well-Being	10.18	2.25	2.1/14.7	−0.31 **	——		
3. Anxiety	5.83	4.71	0/21	0.37 **	−0.43 **	——	
4. Depression	8.49	5.44	0/30	0.39 **	−0.54 **	0.72 **	——

Note. M = mean; SD = standard deviation. ** *p* < 0.01.

**Table 2:** Model fit indices for the latent profile analysis of adolescent smartphone addiction risk.

Number of Profiles	AIC	BIC	aBIC	Entropy	LMR	BLRT	Distribution
2	70,742.238	70,918.223	70,819.732	0.821	0.004	0.000	58.2%/41.8%
**3**	**68,241.557**	**68,479.988**	**68,346.549**	**0.868**	**0.000**	**0.000**	**30.9%/52.7%/16.4%**
4	67,487.286	67,788.164	67,619.776	0.841	0.118	0.000	22.7%/44.4%/25.6%/7.3%
5	66,856.912	67,220.236	67,016.900	0.855	0.054	0.000	22.8%/43.0%/20.1%/7.4%/6.7%

Note. Boldface indicates the selected model. AIC = Akaike information criterion; BIC = Bayesian information criterion; ABIC = adjusted Bayesian information criterion; LMR = Lo–Mendell–Rubin adjusted likelihood ratio test; BLRT = bootstrap likelihood ratio test.

**Table 3:** Multinomial logistic regression of demographic predictors of latent profiles of adolescent smartphone addiction risk.

Predictor	The Low-Risk Group		The High-Risk Group	
	β (95% CI)	OR (95% CI)	β (95% CI)	OR (95% CI)
Sex	−0.099 [−0.315, 0.117]	0.906 [0.730, 1.123]	0.152 [−0.122, 0.426]	1.165 [0.885, 1.531]
Grade	0.382 *** [0.308, 0.457]	1.465 *** [1.359, 1.578]	0.564 *** [0.466, 0.662]	1.758 *** [1.594, 1.939]
Age	0.318 *** [0.249, 0.387]	1.374 *** [1.283, 1.472]	0.481 *** [0.393, 0.569]	1.618 *** [1.481, 1.766]
SSES	−0.196 *** [−0.282, −0.110]	0.822 *** [0.754, 0.896]	−0.200 *** [−0.320, −0.080]	0.819 *** [0.726, 0.923]

Note. The well-adjusted smartphone use group was the reference category. OR = odds ratio; CI = confidence interval; SSES = subjective socioeconomic status (MacArthur Scale, 1–10; a single-item measure; higher scores = higher perceived social standing). *** *p* < 0.001.

**Table 4:** Differences in mental health outcomes among latent profiles of adolescent smartphone addiction.

Outcome Variable	C1	C2	C3	Wald χ^2^ (df = 2)	Pairwise Comparisons	Hedges’ g [95% CI]
Well-being (M ± SE)	11.184 ± 0.096	9.967 ± 0.065	9.008 ± 0.123	208.364 ***	C1 > C2 ***; C1 > C3 ***; C2 > C3 ***	C1 vs. C2: 0.53 [0.43, 0.63]; C1 vs. C3: 0.91 [0.78, 1.05]; C2 vs. C3: 0.45 [0.33, 0.57]
Anxiety (M ± SE)	3.675 ± 0.169	6.053 ± 0.134	9.064 ± 0.310	265.463 ***	C1 < C2 ***; C1 < C3 ***; C2 < C3 ***	C1 vs. C2: −0.55 [−0.65, −0.45]; C1 vs. C3: −1.12 [−1.26, −0.98]; C2 vs. C3: −0.62 [−0.74, −0.50]
Depression (M ± SE)	5.687 ± 0.200	8.839 ± 0.154	12.539 ± 0.324	352.628 ***	C1 < C2 ***; C1 < C3 ***; C2 < C3 ***	C1 vs. C2: −0.61 [−0.71, −0.51]; C1 vs. C3: −1.24 [−1.38, −1.10]; C2 vs. C3: −0.67 [−0.79, −0.55]

Note. C1 = the well-adjusted use group; C2 = the low-risk group; C3 = the high-risk group; M = mean; SE = standard error; Wald χ^2^ = Wald chi-square test; df = degrees of freedom. *** *p* < 0.001.

## Data Availability

The raw data are not publicly available due to the inclusion of minors and ethical constraints. Data can be obtained by contacting the corresponding author with a justified research request.
